# Imaging in inflammatory bowel disease 2025: ECCO-ESGAR-ESP-IBUS diagnostic and monitoring recommendations with MRI and intestinal ultrasound in treat-to-target strategies

**DOI:** 10.1186/s13244-026-02312-6

**Published:** 2026-05-21

**Authors:** Igor Vlašiček, Ann Katrin Kaufmann-Bühler, Marijan Pušeljić, Anselm Schlemmer, Michael Fuchsjäger, Emina Talakić

**Affiliations:** Department of Radiology, Medical University of Graz, 8036 Graz, Austria

**Keywords:** Review, IBD, Multimodality, Guidelines

## Abstract

**Abstract:**

The 2025 ECCO-ESGAR-ESP-IBUS multisociety guidelines mark a paradigm shift in IBD management, positioning imaging as a central element of the treat-to-target strategy. This critical review analyzes these updates from a radiological perspective. Magnetic resonance enterography (MRE) and intestinal ultrasound (IUS) are now established as co-first-line modalities for diagnosis and monitoring, reflecting their proven accuracy and safety. Evidence from trials such as METRIC, TRUST-UC, and PISA-II demonstrates that cross-sectional imaging reliably detects disease activity, complications, and therapeutic response, enabling proactive, non-invasive disease control. The guidelines promote early imaging-based assessment and incorporate transmural healing as an achievable therapeutic target. However, practical barriers remain, including limited access to MRE, operator dependence on IUS, and heterogeneity in the definition of transmural healing and fibrosis. Implementing standardized protocols and structured training is essential to realize the guidelines’ vision. By positioning imaging at the core of IBD care, the 2025 guidelines transform radiology from a diagnostic adjunct to a strategic driver of precision therapy.

**Critical relevance statement:**

Cross-sectional imaging, particularly MRE and IUS, has become indispensable for comprehensive IBD assessment. The 2025 ECCO-ESGAR-ESP-IBUS guidelines integrate imaging into every phase of patient management, underscoring its value for diagnosis, monitoring, and achieving transmural remission. This shift requires structured training and harmonization across Europe.

**Key Points:**

- MRE and IUS have become co-first-line modalities for IBD diagnosis and follow-up with imaging now embedded within the treat-to-target framework, emphasizing early response and transmural healing.
- MRE is preferred for baseline staging and complex complications; IUS excels in dynamic, point-of-care monitoring.
- Standardization and training remain major barriers to implementation.

**Graphical Abstract:**

**Figure:**
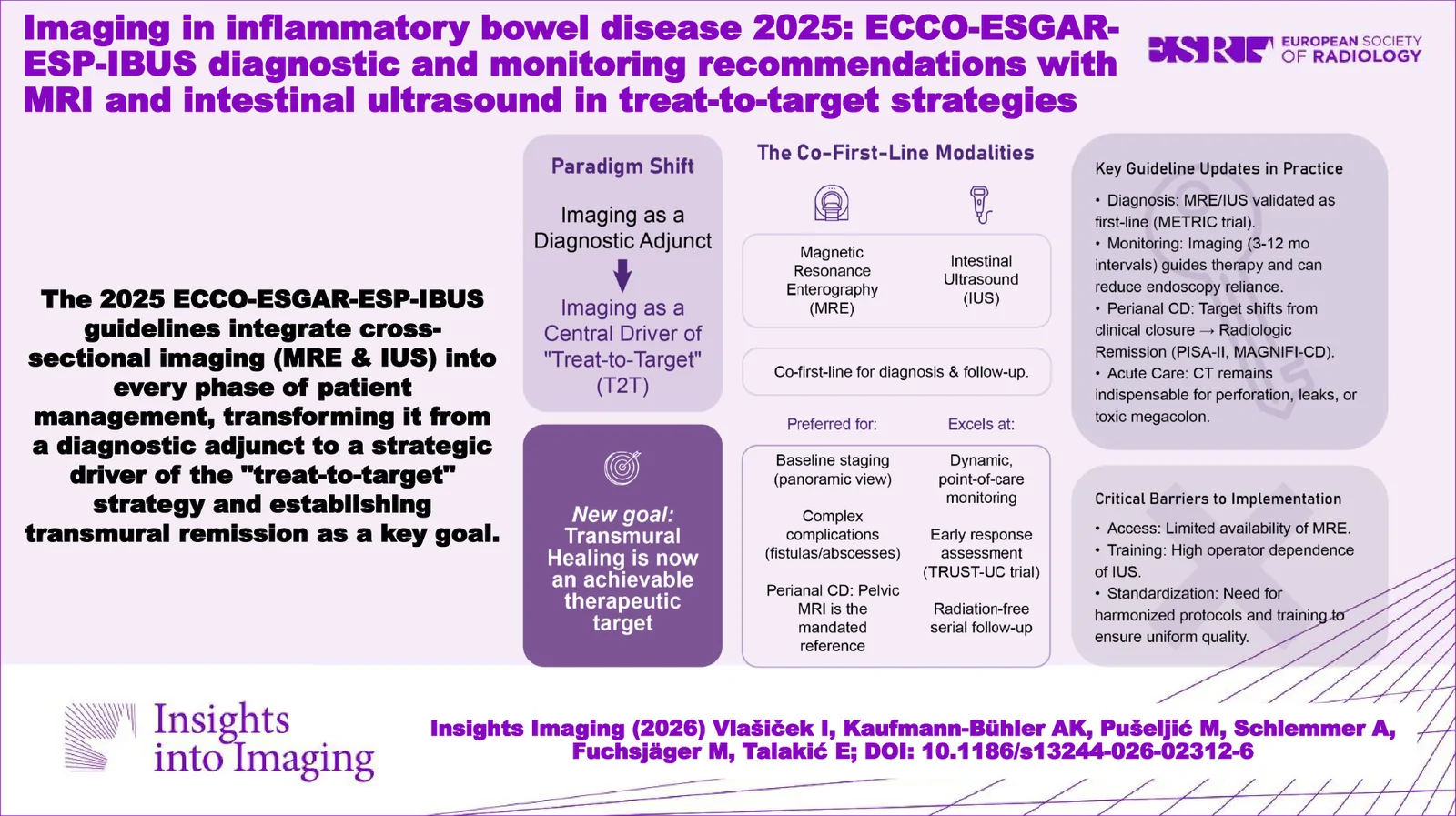

## Introduction

Inflammatory bowel disease (IBD) management has evolved from symptom control toward a structured, outcome-driven “treat-to-target” approach [1]. The 2025 ECCO-ESGAR-ESP-IBUS multisociety guidelines consolidate this philosophy, integrating radiology, gastroenterology, and pathology into a unified diagnostic and monitoring framework [2]. The principal goal is sustained, deep remission, defined not only by clinical and endoscopic improvement but also by transmural healing.

Endoscopy remains the reference standard for mucosal assessment, yet its invasiveness and inability to visualize the entire small bowel limit its applicability for longitudinal monitoring [3]. Cross-sectional imaging, magnetic resonance enterography (MRE), intestinal ultrasound (IUS), and computed tomography (CT) overcome these constraints by visualizing both mural and extramural disease, offering a non-invasive window into inflammation, fibrosis, and complications [3].

The 2025 guidelines mark the first time that MRE and IUS are formally recognized as co-first-line modalities for diagnosis, disease monitoring, and treatment evaluation [2]. This review critically evaluates the new imaging recommendations, analyzing evidence strength, implementation feasibility, and future research needs.

## Diagnostic role of cross-sectional imaging

### From adjunct to first-line modality

The ECCO-ESGAR-ESP-IBUS 2025 guidelines elevate both MRE and IUS from complementary tools to first-line investigations for suspected or established IBD [2]. High-level evidence, notably the METRIC trial, demonstrated excellent diagnostic performance for detecting the presence of active small-bowel Crohn’s disease (MRE sensitivity 97%, specificity 96%; IUS 92%, 84%), with a more pronounced difference in terms of assessing disease extent (MRE sensitivity 80%, specificity 95%; IUS 70%, 81%). [4]. This finding validates their equivalence for identifying active inflammation, while MRE retains a modest advantage for defining disease extent and extramural manifestations [5]. For initial staging, the guidelines recommend MRE as the preferred examination because of its reproducibility and panoramic visualization of the abdomen and pelvis. However, IUS remains strongly endorsed when MRI availability is limited or patient tolerance is a concern. Importantly, the 2025 update introduces a pragmatic compromise: establishing an IUS baseline at diagnosis allows subsequent comparison during therapy monitoring [2, 6].

## Clinical significance of accuracy differences

The roughly 10% higher sensitivity of MRE compared with IUS for the extent of small-bowel disease has uncertain clinical relevance. If therapeutic decisions depend primarily on the presence of disease rather than its exact length, IUS may suffice for many patients. Nevertheless, studies by Lang et al and Greener et al show that MRE reclassified disease phenotype or extent in up to 25% of cases, occasionally prompting treatment modification [7, 8]. Long-term outcome studies are still lacking, underscoring a need for research that correlates imaging-driven re-classification with patient prognosis.

## Integration with endoscopy and biomarkers

Modern IBD assessment relies on a triad of endoscopy, biomarkers, and imaging. Fecal calprotectin and C-reactive protein provide biochemical context but lack spatial resolution. MRE and IUS bridge this gap, correlating strongly with endoscopic activity indices such as SES-CD and UCEIS [3, 9]. Their ability to visualize mural hyperenhancement, bowel-wall thickening, and vascularity extends diagnostic precision beyond mucosal disease.

## Advantages of non-ionizing, patient-centered imaging

Both MRE and IUS offer radiation-free evaluation, an essential consideration for young patients requiring lifelong surveillance. MRE provides superior soft-tissue contrast and standardized scoring systems (MaRIA and sMaRIA) [10–12], while IUS enables real-time, bedside assessment and rapid decision-making [13]. This complementarity supports the guideline’s emphasis on tailoring modality choice to clinical context, expertise, and resource availability rather than enforcing a single algorithm [2].

## Monitoring and treat-to-target imaging

### Early response assessment

The 2025 multisociety guidelines place cross-sectional imaging at the center of the treat-to-target strategy, defining clear time points for evaluating therapeutic response [2]. In ulcerative colitis, IUS is endorsed as an integral element of early response assessment, equivalent to endoscopy within 12 weeks after treatment initiation or optimization.

This recommendation stems from evidence that early sonographic normalization of bowel-wall thickness (BWT) strongly correlates with endoscopic and clinical remission. The TRUST-UC study demonstrated a high correlation between normalization of bowel wall thickness (BWT) and clinical response as early as 2 weeks after starting treatment, with around 40% of the patients showing a reduction of BWT in the descending or sigmoid colon [14].

For Crohn’s disease, prospective trials by Ripollés et al and Kucharzik et al demonstrated a correlation between IUS response and biochemical and clinical response as early as 12 weeks after initiating treatment, demonstrating that sonographic improvement anticipates sustained remission at the 1-year mark [13, 15].

While these data validate IUS as a sensitive, rapid, and patient-friendly biomarker, the guidelines correctly note that all studies used surrogate endpoints. Randomized evidence showing superior outcomes with IUS-guided management versus conventional care is still lacking. Future interventional trials are therefore warranted.

## Cross-sectional imaging in longitudinal monitoring

The updated framework integrates MRE and IUS into a continuous monitoring schedule. Endoscopic reassessment is now postponed to within 12 months, reflecting confidence that non-invasive imaging can safely guide intermediate therapeutic decisions. Both modalities quantify transmural improvement more comprehensively than colonoscopy, which evaluates only the mucosa. Serial IUS or MRE examinations are recommended at 3–6-month intervals after therapy initiation and every 6–12 months during remission [2, 13].

## Transmural healing as a therapeutic target

One of the most transformative changes in the 2025 guidelines is the formal acceptance of transmural healing as an achievable and desirable therapeutic goal [2]. Previously considered aspirational, this concept is now supported by multiple prospective studies demonstrating its association with durable remission and reduced hospitalization. In Crohn’s disease, transmural healing - defined by normalized BWT (< 3 mm), absent Doppler signal, and restored bowel stratification on IUS or by normal MaRIA/sMaRIA scores on MRE, is linked to the lowest relapse and surgery rates [16–18]. A recently published international consensus provides the first harmonized definition of ultrasound response for CD and UC, as well as ultrasound remission in CD, thus enabling better comparison between studies going forward [19]. A consensus definition of US remission in UC, as well as definitions pertaining to MRI, are missing.

Despite the progress in imaging, actually achieving full transmural healing remains challenging. With studies showing that complete transmural healing is only achievable in 20–40% of patients with current therapies [20], radiologists can be expected to frequently encounter “partial response” without reaching the desired target. This poses the question of how to quantify and communicate the clinical significance of these incremental changes.

With the newly central role of radiology in assessing therapeutic success in IBD comes the urgent need to develop and validate robust definitions to facilitate both further research and allow for better clinical implementation of cross-sectional imaging.

## Therapy de-escalation and relapse prediction

The 2025 update recommends closer follow-up in the critical early period after treatment de-escalation or withdrawal, especially in the 1st year, as studies have shown relapse rates of 29–50% [2, 21–24]. The authors concede that optimal intervals for monitoring are not yet established, but consider a IUS performed 3 months after treatment withdrawal as feasible [2]. This proactive approach supports individualized, risk-adapted management.

## Proactive surveillance in remission

For patients with CD in sustained clinical and biochemical remission, the guidelines newly recommend periodic imaging every 6–12 months [2]. IUS is preferred for its convenience and cost-effectiveness, whereas MRE remains indicated when disease activity is uncertain or small-bowel visualization is suboptimal. In ulcerative colitis, the recommendation for monitoring is less strong, marking it as an emerging, adjunct tool alongside clinical and biochemical indicators [2], drawing on a study by Maeda et al, which demonstrated high accuracy of the Milan ultrasound criteria (MUC) in predicting a clinical relapse, surpassing other IUS indicators such as BWT and bowel wall blood flow alone [25].

## Imaging of complications and postoperative assessment

### Strictures and fibrostenotic disease

For stricturing disease, the fundamental recommendations remain unchanged - MRE and IUS are reaffirmed as the primary modalities outside of the acute setting. The guidelines maintain that no imaging technique can accurately quantify the degree of fibrosis, but rate MRE as superior to IUS or computed tomography enterography (CTE) for determining the extent of fibrosis [2]. The new guidelines now explicitly warn about the low sensitivity of cross-sectional imaging for small bowel adenocarcinoma in patients with CD, based on a systematic review that found only 11% had observable radiological features [26].

The quantification of fibrosis remains an issue, with the guidelines reemphasizing the limitations of current cross-sectional modalities to accurately quantify it [2]. This creates a gap, with clinicians being encouraged to treat a pathological process (fibrosis) that they cannot accurately and non-invasively monitor. The main question is often not the exact percentage of stenosis, but the differentiation between predominantly inflammatory and fibrotic strictures - a distinction which puts the patient on a path towards medical (inflammatory) or endoscopic/surgical (fibrotic) management [27]. Traditional cross-sectional imaging methods fall short of this goal, but advanced techniques such as elastography or diffusion-weighted MRI sequences still provide future perspectives - the implementation of these techniques in routine practice requires further evaluation to determine cutoff values, sensitivity and specificity [28].

## Fistulas and abscesses

Cross-sectional imaging remains essential for the diagnosis and follow-up of penetrating disease. MRE provides a comprehensive visualization of fistulous tracts, abscesses, and hidden retroperitoneal extensions. IUS and transabdominal contrast-enhanced ultrasound can identify superficial or interloop fistulas, whereas CT maintains a role in emergency settings for rapid abscess detection [2, 29]. A key update in the 2025 document is the multi-tiered imaging approach: if the first modality is inconclusive and clinical suspicion persists, a second cross-sectional study should be performed using an alternative technique [2]. This pragmatic recommendation aims to reduce missed complications without exposing patients to unnecessary radiation.

## Perianal Crohn’s disease

Perhaps the most progressive aspect of the new guidelines is the shift from clinical closure to radiologic remission as the therapeutic target in perianal Crohn’s disease. Driven by the PISA-II trial and validation of the MAGNIFI-CD index, pelvic MRI is now mandated as the reference modality [30, 31]. Re-assessment is recommended within 6 months of any treatment change, as MRI findings of fibrosis and absence of abscess predict long-term closure better than external inspection. Van Rijn et al showed that a MAGNIFI-CD score ≤ 6 (out of 25) predicts long-term closure with 91% specificity and 87% sensitivity [31]. When MRI is unavailable or contraindicated, transrectal ultrasound (TRUS) can serve as an alternative with comparable sensitivity (≈ 87%) but lower specificity (43%) [14]. Transperineal ultrasound (TPUS) is a tertiary option, although evidence for its ability to assess deep healing remains limited [12].

## Post-surgical complications and pouch disorders

Postoperative leaks and intra-abdominal collections remain best evaluated with contrast-enhanced CT, owing to its availability and high diagnostic speed [2, 29]. A relevant change concerns the evaluation of ileo-anal pouches. The 2019 guidelines present endoscopy as the primary tool in the diagnosis of pouchitis and associated complications [6]. The updated guidelines slowly introduce cross-sectional imaging into the diagnostic toolkit, suggesting that cross-sectional modalities “might be feasible alternatives”, especially in suspected cases of extramural pouch complications. This decision is justified by the sparse data on imaging in pouchitis and the lack of studies comparing different modalities, which presents a high degree of uncertainty about the reference standard [2], while also taking into account the intrinsically superior ability of cross-sectional imaging to detect extramural complications.

## Computed tomography in acute settings

Despite the preference for radiation-free modalities, computed tomography (CT) remains indispensable in emergency IBD care. The 2025 ECCO-ESGAR-ESP-IBUS guidelines clearly restrict CT to acute or complicated scenarios where rapid, comprehensive evaluation is required [2]. In suspected toxic megacolon, CT has replaced plain abdominal radiography as the first-line examination because of its superior sensitivity for colonic dilatation, mural thinning, and impending perforation [2]. Similarly, CT angiography (CTA) is now explicitly endorsed for the detection of acute gastrointestinal bleeding, particularly from small-bowel sources. Reported sensitivity and specificity reach 89% and 92%, respectively [2]. CT also remains the method of choice for postoperative leaks, perforations, or intra-abdominal sepsis, where speed and availability outweigh radiation concerns [29]. Overall, CT remains an indispensable tool in the acutely unwell patient, where its speed, availability, and accuracy outweigh radiation concerns.

## Integrating imaging into routine clinical practice

While the 2025 guidelines provide a framework for the use of cross-sectional imaging, transferring the recommendations into practice requires consideration of their individual strength and weaknesses, as well as the evidence supporting their utility in specific clinical scenarios, particularly when pursuing deeper therapeutic targets like transmural healing.

Presently, the debate over “MRE vs IUS” is shifting towards a discussion of their complementary roles. The optimal strategy is not to choose the “superior” modality but to use their individual strengths to inform clinical decisions at various points in the patient’s journey.

The main strength of MRE lies in the ability to provide a comprehensive, objective, and reproducible assessment of the entire abdomen and pelvis in a single examination [2]. The excellent soft tissue contrast enables detailed visualization of the bowel wall and extramural tissues, making it the ideal tool for several important tasks. Firstly, as established by the METRIC trial and reinforced in the 2025 guidelines, MRE is the superior tool for initial staging, providing a “road map” of disease extent and phenotype [2, 4], providing exceptional value in terms of initial risk stratification and treatment planning.

Second, MRE excels in the evaluation of complex penetrating complications, particularly deep or retroperitoneal fistulae and abscesses that may be inaccessible to IUS [2].

Lastly, the availability of validated and reproducible activity scores, such as the Magnetic Resonance Index of Activity (MaRIA), and its more clinically feasible simplified version (sMaRIA), provides the tools necessary for establishing an objective baseline and measuring treatment response in the context of clinical trials, and is now explicitly endorsed by the updated guidelines [10–12].

Despite these clinical advantages, patient tolerability remains a significant practical barrier to successful examination completion. While the imaging procedure itself is safe, the required ingestion of oral contrast agents, such as mannitol or polyethylene glycol, regularly induces gastrointestinal side effects. Patients frequently report symptoms including feelings of fullness, abdominal pain, spasms, and diarrhea, citing the oral preparation as the least acceptable part of MRE [32].

The primary advantage of IUS is not its panoramic view, but its usefulness as a dynamic monitoring tool. The non-invasive nature, lack of need for preparation, low cost, and wide availability make it the ideal tool for point-of-care use by the treating gastroenterologist or dedicated sonographer, allowing for real-time assessment and immediately informing clinical decisions [29]. A patient presenting with worsening symptoms can be rapidly evaluated with IUS, allowing for immediate differentiation between a disease flare and non-inflammatory causes (e.g., irritable bowel-like symptoms), with the therapy being adjusted as appropriate [2]. A study by St-Pierre et al found that by using point-of-care IUS, 85% of patients with symptoms of active IBD presenting to an acute IBD clinic avoided urgent endoscopy [33]. This capability perfectly aligns with the “treat-to-target” strategy. The 2025 guidelines’ recommendation for a 12-week response assessment is largely driven by the practicality and proven accuracy of IUS. A growing body of evidence demonstrates that early changes on IUS, such as reduction in BWT and color Doppler signal, are predictive of long-term endoscopic response and favorable clinical outcomes, with the Milan ultrasound criteria and the UC-IUS (ulcerative colitis intestinal ultrasound score) being validated using endoscopy [12, 25, 34, 35].

The current evidence supports a complementary role of MRE at diagnosis for comprehensive staging and phenotyping, with subsequent monitoring with IUS at frequent intervals (e.g., 3–6 months) to track response to therapy. MRE can be used in situations where IUS is inconclusive or fails to visualize suspected disease, or when a comprehensive re-evaluation is needed prior to a major therapeutic decision (Table 1).

**Table 1:** Recommended imaging modalities for major IBD-related complications

Clinical scenario	First-line modality	Alternative/adjunct	Key rationale
Fibrostenotic stricture	MRE	IUS	MRE maps extent; IUS for follow-up; elastography under evaluation
Fistula/abscess (abdominal)	MRE	CT (acute), CE-US	MRE for chronic/complex; CT in emergency
Perianal disease	MRI pelvis	TRUS/TPUS	MRI preferred; TRUS when MRI unavailable
Postoperative leak	CT with contrast	-	Fast, sensitive for extraluminal gas/fluid
Pouch complications	Pouchoscopy with biopsy	MRE/TPUS/CT	Imaging detects extramural pathology

*MRE* magnetic resonance enterography, *IUS* intestinal ultrasound, *CE-US* contrast-enhanced ultrasound, *TRUS* transrectal ultrasound, *TPUS* transperineal ultrasound

The 2025 guidelines revisit the concept of transmural healing, backed up by high-quality data published in the interim. In 2019, it was an aspirational concept, a logical, but still unproven endpoint rooted in the transmural nature of CD, but lacking a formal recommendation to treat to a target of transmural healing. The 2025 guidelines establish transmural healing as an achievable therapeutic target, associated with better outcomes [2]. This decision is based on high-quality data, which has demonstrated that transmural healing is superior to mucosal healing alone in predicting long-term remission [16, 36]. Data from other large studies and literature reviews published in recent years conclusively point to transmural healing as a desirable target in treating patients with CD [17, 18]. With this new data, the guidelines have matured from tentative suggestion to active recommendation, advocating for proactive monitoring even in asymptomatic patients, with the goal of detecting and managing subclinical inflammation. This reflects a significant shift in the treatment strategy, making the assessment and healing of the entire bowel wall a key driver of therapeutic decisions. The goal is no longer just to heal the mucosa, but to achieve a deeper, more durable remission in the hopes of preventing disease progression and altering the natural history of the disease. While challenges in definition and achievability remain, this change in guidance marks a new era in IBD care.

## Implementation challenges and training needs

Translating these ambitious imaging recommendations into routine practice poses significant logistical and educational challenges.

MRE requires high-field scanners, optimized protocols, and radiologists experienced in abdominal imaging, resources concentrated mainly in tertiary centers [2]. Lack of standardized acquisition and reporting parameters hinders comparability among institutions. Adoption of simplified MaRIA scoring and structured reporting templates is encouraged to harmonize communication between radiologists and clinicians [10–12].

The principal obstacle to IUS implementation is its operator dependence. The guidelines reference the IBUS training curriculum, recommending a minimum of 80-200 supervised cases for competency [12]. Yet few centers can currently provide such training volume. Inter-observer variability can be high, particularly for novice operators, with the acquisition of necessary skills made more difficult by the lack of standardized curricula or certification in many countries [37]. Even when a standardized curriculum (such as the IBUS curriculum) [12] exists, training a sufficient number of radiologists or gastroenterologists to meet clinical demand remains a challenge, which could delay the implementation of IUS in clinical practice for years to come. On the other hand, a rushed education poses the risk of premature substandard adoption, leading to inaccurate diagnoses and misguided clinical decisions.

These challenges risk creating a two-tiered system of care, with patients in developing countries and rural areas not having access to frequent, non-invasive monitoring, and instead having to rely on more invasive or less frequent assessment, potentially leading to delayed treatment and worse short- and long-term outcomes.

## Economic and practical considerations

The economic dimension of imaging-intensive management cannot be overlooked. Although MRE entails substantially higher unit cost and longer scheduling delays, IUS offers a cost-effective, point-of-care alternative. A UK cost-analysis reported mean pathway costs of £78.86 (~ €92) for IUS versus £375.35 (~ €438) for MRE, with the time from referral to treatment halved in the IUS arm (46 vs. 91 days) [38].

However, the broader financial argument favors imaging when considering its ability to prevent complications and hospitalizations through early detection and tailored therapy. With studies demonstrating the improvement in disease outcomes under tight monitoring, balancing short-term expenditure against long-term savings and patient quality of life appears a pragmatic choice.

Reimbursement frameworks must evolve to reflect the true value of high-quality imaging and multidisciplinary collaboration. Establishing dedicated “IBD imaging clinics,” where radiologists and gastroenterologists perform joint assessments, has already demonstrated improved efficiency and patient satisfaction in pilot programs [2].

## Future directions

### Toward quantitative and AI-assisted imaging

The next frontier in IBD imaging lies in standardization, quantification, and automation.

Artificial-intelligence-based segmentation of bowel loops and radiomics feature extraction from MRE or IUS datasets show strong potential for objective inflammation grading and fibrosis prediction [39]. Studies have demonstrated agreement rates of 75% (for the bowel lumen) and 81% (for the bowel wall) with automated segmentation [5].

Integration of AI into reporting workflows could reduce inter-observer variability, enhance reproducibility, and allow real-time decision support during multidisciplinary IBD boards.

## Fibrosis quantification and novel biomarkers

Accurate non-invasive differentiation between inflammatory and fibrotic strictures remains an unmet need.

Promising methods include magnetization-transfer MRI, T1/T2 mapping, diffusion kurtosis, and shear-wave ultrasound elastography [27, 28]. Multi-parametric models combining imaging biomarkers with serum and fecal indices are under evaluation in multicenter trials.

Future guidelines are expected to include quantitative thresholds for fibrosis and standardized cut-off values for transmural healing.

## Harmonization and education

The new guidelines, more than ever, highlight the importance of global harmonization. Developing unified training curricula and shared image repositories will accelerate adoption, while structured reporting templates, integrated in PACS or AI-assisted platforms, will ensure consistent communication.

Cross-disciplinary education, with radiologists teaching gastroenterologists basic IUS skills and vice versa, exemplifies the collaborative spirit envisioned by the new guidelines.

## Conclusion

The 2025 multisociety guidelines redefine IBD imaging as a central, proactive component of disease management rather than a confirmatory tool.

MRE and IUS are now co-first-line modalities for diagnosis and longitudinal assessment, seamlessly integrated into the treat-to-target strategy.

Radiology’s role extends beyond detection - to quantification, prognosis, and therapy guidance.

Successful implementation will depend on equitable access, standardized training, and evidence that imaging-driven decisions improve long-term outcomes.

As artificial intelligence, elastography, and radiomics mature, cross-sectional imaging will continue to evolve from a descriptive to a truly quantitative discipline, shaping the future of personalized care in IBD.

## Data Availability

Not applicable to this article as no new datasets were generated or analyzed during the current study.

## Abbreviations

BWT: Bowel wall thickness
IBD: Inflammatory bowel disease
IUS: Intestinal ultrasound
MRE: Magnetic resonance enterography
MaRIA: Magnetic Resonance Index of Activity
