# Correction: Imaging in inflammatory bowel disease 2025: ECCO-ESGAR-ESP-IBUS diagnostic and monitoring recommendations with MRI and intestinal ultrasound in treat-to-target strategies

**DOI:** 10.1186/s13244-026-02432-z

**Published:** 2026-10-29

**Authors:** Igor Vlašiček, Ann Katrin Kaufmann-Bühler, Marijan Pušeljić, Anselm Schlemmer, Michael Fuchsjäger, Emina Talakić

**Affiliations:** Department of Radiology, Medical University of Graz, 8036 Graz, Austria

**Correction to: Insights into Imaging** (2026) 17:137https://doi.org/10.1186/s13244-026-02312-6published online 21 May 2026

In this article, the authors Ann Katrin Kaufmann-Bühler, Marijan Pušeljić, Anselm Schlemmer, and Michael Fuchsjäger, all at the affiliation ‘Department of Radiology, Medical University of Graz, 8036 Graz, Austria’, were missing from the author list. The added authors declare no competing interests. ‘The other authors contributed to the critical revision and editing of the manuscript.’ has been added to the Author Contributions section.

The original article has been corrected.

